# Genetic Structure Along Temperature Gradient in a Recent Invader *Cardamine hirsuta* in Eastern Japan

**DOI:** 10.1002/ece3.74480

**Published:** 2026-10-09

**Authors:** Bo O. Zhang, Yiheng Shen, Hiroshi Ozaki, Naofumi Yoshida, Shin‐Ichi Morinaga, Onno Muller, Kouki Hikosaka

**Affiliations:** ^1^ Graduate School of Life Sciences Tohoku University Sendai Japan; ^2^ Translational Research Support Office National Cancer Center Hospital East Kashiwa Japan; ^3^ Department of Natural & Environmental Science Teikyo University of Science Tokyo Japan; ^4^ Institute for Bio‐and Geosciences, IBG‐2: Plant Sciences Research Center Jülich GmbH Jülich Germany

**Keywords:** *Cardamine hirsuta*, climate association, distribution modeling, genetic structure, invasive plant species

## Abstract

Invasive species often spread rapidly, which appears discrepant because species distribution is often limited by environmental conditions. Even in widely distributed species, there are differences among genetic clusters along environmental gradients, suggesting that the environmental range of one genetic group is limited but extended distribution is possible through genetic differentiation. *Cardamine hirsuta* is native to Europe, invaded Japan in the 1970s, and is now found throughout Japan. To understand how *C. hirsuta* has rapidly spread across a climatic gradient in eastern Japan, where there is a large temperature gradient along the latitude, we characterized its genetic structure and addressed three hypotheses: (1) a genetic cluster is widely distributed because it favors a broad range of environmental conditions, (2) different genetic clusters are distributed across distinct environmental conditions because of different environmental preferences, and (3) admixed plants between different genetic clusters fill gaps where original genetic clusters are less adapted. We analyzed 82 individuals collected from 29 sites, and *ADMIXTURE* analysis showed the lowest cross‐validation error at *K* = 4. Cluster 2 was widely distributed in eastern Japan, whereas Cluster 3 was found in northern areas and Cluster 1 was found in southern areas. Individuals with mixed ancestry were observed, particularly in regions where the distributions of genetic clusters overlapped. The potential distributions of different clusters, which were associated mainly with the temperature gradient, overlapped without obvious gaps, providing no evidence that individuals with mixed ancestry preferentially occurred in gaps between the potential distributions of the original genetic clusters. We conclude that genetic clusters within an invasive species can show contrasting climatic associations, while some clusters may occur across a comparatively broad climatic range. Considering such intraspecific genetic structure may provide additional information for assessing potential range expansion and contribute to more effective monitoring of ongoing invasions.

## Graphical abstract

*Cardamine hirsuta* has rapidly expanded across a broad climatic gradient in eastern Japan despite its recent introduction. By analyzing genetic structure and climatic associations, we identified multiple genetic clusters with distinct distribution patterns and environmental associations, as well as admixture among clusters in overlapping regions. Our results show that some genetic clusters occur across a comparatively broad climatic range, highlighting the value of considering intraspecific genetic structure when assessing the range expansion of invasive species.

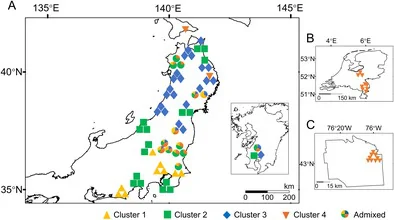

## 1 Introduction

Invasion of alien species has emerged as a considerable issue in recent years because it often has adverse effects on the survival of native species and is detrimental to local ecosystems (Keller et al. 2011; Marbuah et al. 2014; Kovács‐Hostyánszki et al. 2022). In many cases, invasive species exhibit rapid spread and successful establishment within a new environment. For instance, *Lythrum salicaria* , introduced to the United States of America (USA) in the early 19th century, spread with high speed and was present throughout the country by 2017 (Zimdahl and Cynthia 2018). *Spartina alterniflora* invaded and expanded in China, reaching 55,181 ha within 35 years (Zhang et al. 2017). *Ageratina adenophora* , one of the worst invasive plants, has increased its distribution in the southern subtropical areas of China at an average rate of 20 km per year (Wang and Wang 2006). Understanding the process underlying such rapid expansion is therefore important for invasion biology.

Climate factors including temperature and precipitation play a key role in determining the distribution of plant species (Finch et al. 2021). Suitable temperatures for distribution differ considerably among species. For example, *Stipa purpurea*, a dominant alpine herb in the Tibetan Plateau, occurs in regions with a mean annual temperature (MAT) of 0°C–3°C (Ma et al. 2022); *Abies grandis* is distributed in regions with 6°C–10°C MAT (Skorupski et al. 2011); and *Bertholletia excelsa*, a long‐lived South American tree, is distributed at 23.5°C–27.6°C MAT (Thomas et al. 2014). The breadth of the suitable temperature range also differs among species. For example, *Nidularium minutum* can be distributed between 2°C and 30°C (Carvalho et al. 2013), whereas *Annona muricata* is distributed only between 25°C and 28°C (Gavamukulya et al. 2017; Coria‐Téllez et al. 2018). Even within widely distributed species, genetic differentiation exists among populations that inhabit diverse environmental conditions (Ahrens et al. 2019; Li et al. 2020; Li, Xia, et al. 2024). For example, *Arabidopsis thaliana* , a popular model plant with a wide distribution throughout the world, has genetic and phenotypic differentiation along environmental variables of its original habitat, such as temperature and precipitation (e.g., Hopkins et al. 2008; Burghardt et al. 2016; Ozaki et al. 2018; Adams III et al. 2023). These findings suggest that genetic differentiation may contribute to variation in environmental associations within species, and that the environmental range in which a genetic cluster can exist is limited.

Admixture among genetically differentiated clusters has been proposed as a process that influences species distributions across environmental gradients. In some cases, admixed individuals are observed and filled in habitats where parental clusters are absent or less abundant, indicating that admixture may facilitate the occupation of intermediate environments (e.g., Segarra‐Moragues et al. 2016; Stacy et al. 2016; Muniz et al. 2022). For example, in a study that along an altitudinal gradient, admixed plants were dominant in intermediate zones where neither highland nor lowland ecotypes occurred (Yoshida et al. 2023). Furthermore, admixture may generate heterosis or novel trait combinations that enhance performance under certain environmental conditions by combining genetic backgrounds from different clusters (Hahn and Rieseberg 2016; Li et al. 2018; Shi et al. 2018). For instance, in *Arabidopsis thaliana* , hybrid individuals of different accessions often exhibit heterosis in growth rates (Fujimoto et al. 2012; Mishio et al. 2024). These observations suggest that admixed individuals may occupy ecological space not fully utilized by parental clusters or exhibit competitive advantages under certain conditions. Therefore, examining the occurrence and distribution of admixed individuals is important for understanding their potential role in expanding species distributions.

*Cardamine hirsuta* (Hairy bittercress) is an invasive plant native to Europe that has spread to many regions worldwide, including Australia, the USA, and other countries (Kudoh et al. 2006). The oldest recorded specimen in Japan was collected in 1974, and now it can be found throughout Japan across a latitudinal range from 31° N to 45° N (Kudoh et al. 2006). However, it remains unclear how *C. hirsuta* has expanded across the broad latitudinal and temperature gradient in Japan. It has been shown that there are three different genetic groups of *C. hirsuta* in Japan (Matsuhashi et al. 2016), and the number of lateral stamens among the three groups suggests phenotypic differentiation occurred (Matsuhashi et al. 2012). However, it is still unknown whether the geographical distribution of the three groups was restricted by the different climatic conditions. Furthermore, because Matsuhashi et al. (2016) used a microsatellite marker method, which generates limited genetic resolution, *C. hirsuta* in Japan may have differentiated into more genetic clusters.

Despite climate factors that limit plant distribution, *C. hirsuta* has rapidly expanded across a wide range of environments. Therefore, it is important to understand how *C. hirsuta* has achieved its broad distribution across a climatic gradient. Here, we address three hypotheses. First, a species can be distributed in a broad environmental range if a genotype has an adaptability to the large environmental variation. Thus, we hypothesized that (1) a genetic cluster is widely distributed because it favors a broad range of environmental conditions. Second, broad species distributions can be achieved if genetically differentiated clusters occupy different parts of the environmental gradient. Thus, we hypothesized that (2) different genetic clusters are distributed across distinct environmental conditions because of different environmental preferences. Third, admixture may contribute to broad species distribution when admixed individuals occupy environmental conditions that are not occupied by the original genetic clusters. Thus, we hypothesized that (3) admixed plants between different genetic clusters fill gaps where original genetic clusters are less adapted (Figure 1). To examine these hypotheses, we collected *C. hirsuta* plants from two latitude transects set in eastern Japan, which spanned a broad mean annual temperature (MAT) gradient of approximately −3°C to 18°C, and analyzed their genetic differentiation using the restriction‐site associated DNA sequencing (RAD‐seq). We further applied species distribution models to estimate the environmental range of their potential distribution for each genetic cluster. These analyses improve our understanding of the genetic structure and possible invasion patterns of *C. hirsuta* in eastern Japan.

**FIGURE 1:**
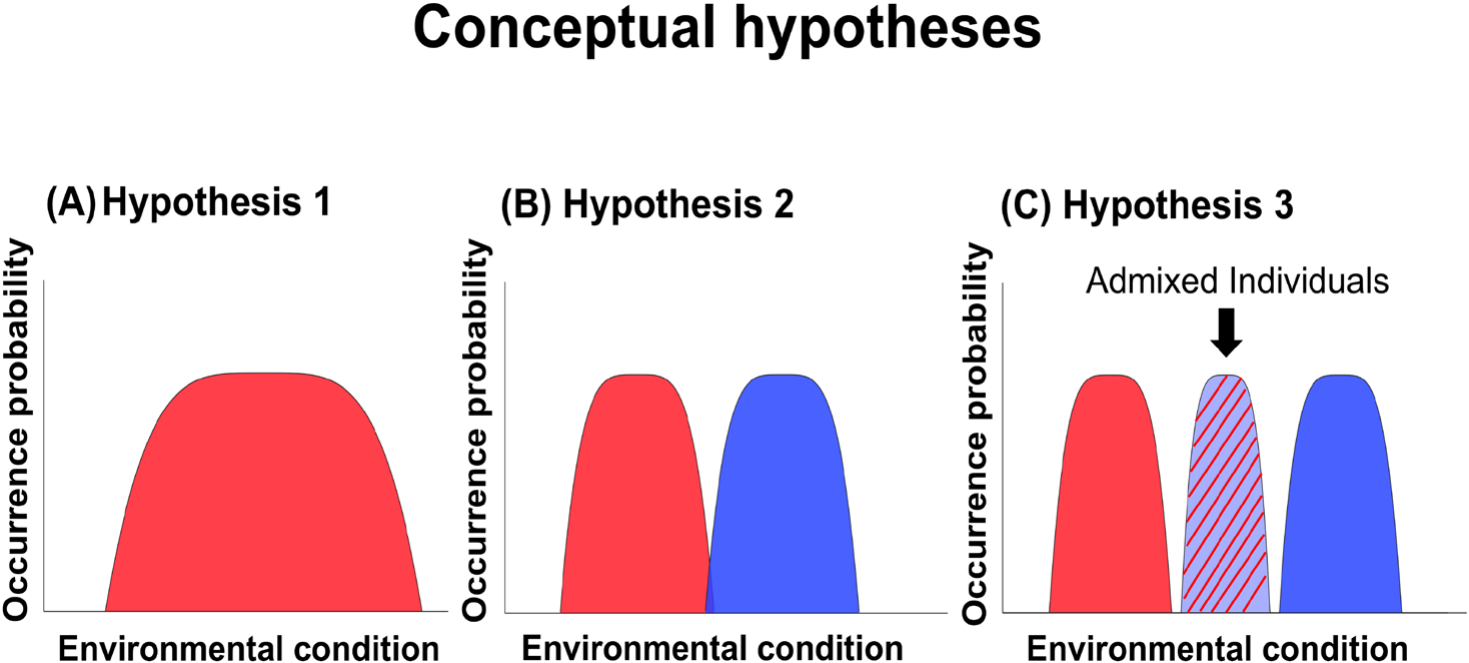
Conceptual hypotheses for the distribution of genetic clusters and admixed individuals across an environmental gradient. (A), (B), and (C) represent Hypotheses 1, 2, and 3, respectively.

## 2 Materials and Methods

### 2.1 Plant Material

*C. hirsuta* is a winter annual herb of the Brassicaceae family (Grime et al. 1988; Rich 1991). It is a common weed found in gardens, turf, and disturbed ground (Hay et al. 2014; Supporting Information S1). This invasive species was first collected in Japan in 1974 and then subsequently appeared sporadically in several prefectures on Japan's mainland, including Shiga, Niigata, Yamagata, Miyagi, and Toyama, in the 1990s (Kudoh et al. 1992). In 2004, the existence of this invasive species was confirmed in Sapporo, Hokkaido (Aida 2004). Over the past few decades, *C. hirsuta* has continued to be discovered in various locations, indicating the gradual expansion of its distribution in Japan (Kudoh 2012; Matsuhashi et al. 2016). In Japan, the germination period usually starts in September (Yatsu et al. 2003), and the flowering period progresses from February to May, and a large number of seeds are produced via auto pollination (Yatsu et al. 2003; Kudoh 2017).

### 2.2 Sampling and Single‐Nucleotide Polymorphism (SNP) Calling

Seeds from 82 individual plants were collected from 29 sites, including Japan, the Netherlands (NL), and the USA. The Japanese samples were collected at 20 sites along the latitude gradient (34°53′–40°55′ N) with two transects set at the east and west sides of eastern Japan in 2014 and were also collected from Kagoshima in 2015 and Hakodate in 2016 (Table 1). In the USA, samples were obtained from Syracuse in 2020, while samples from the NL were collected from four locations in three areas, Herten, Melick, and Maarn in 2020 (Table 1). At each sampling site, seeds were collected from three or more naturally occurring maternal plants. Seeds were stored at room temperature until the experiment. We selected three maternal plants per site. Their seeds were sown and germinated in pots containing commercial soil with fertilizer. One individual per maternal plant was used for DNA extraction.

**TABLE 1:** Location of sampling sites.

Sampling location	Abbreviation	Location site
Kagoshima	KGS	31°32′51.0″N 130°31′44.7″ E
Fujieda	FJD	34°53′14.8″N 138°17′37.6″ E
Awa	AWA	34°55′27.8″N 139°50′14.8″ E
Odawara	ODW	35°17′56.4″N 139°10′07.4″ E
Kimitsu	KMT	35°19′05.9″N 139°55′42.7″ E
Hino	HNO	35°38′46.6″N 139°22′05.2″ E
Tsuchiura	TCR	36°04′23.3″N 140°12′28.2″ E
Maebashi	MBS	36°22′32.6″N 139°02′03.0″ E
Utsunomiya	UTN	36°33′53.7″N 139°58′57.8″ E
Motomiya	MTM	37°30′45.8″N 140°23′42.5″ E
Niigata	NGT	37°51′39.0″N 138°59′50.7″ E
Furukawa	FRK	38°35′31.3″N 140°56′16.6″ E
Atsumi	ATM	38°36′22.3″N 139°35′17.5″ E
Kitakami	KTK	39°14′11.1″N 141°05′39.8″ E
Yurihonnjo	YRH	39°21′36.4″N 140°04′41.7″ E
Joboji	JBJ	40°11′33.0″N 141°10′07.7″ E
Tashiro	TSR	40°16′40.4″N 140°28′24.5″ E
Kuroishi	KRS	40°37′23.7″N 140°37′00.5″ E
Hachinohe	HCH	40°29′11.5″N 141°31′13.3″ E
Aomori	AMR	40°50′09.4″N 140°41′26.8″ E
Arito	ART	40°55′13.9″N 141°11′41.1″ E
Hakodate	HD	41°46′59.2″N 140°46′30.7″ E
US Syracuse	USSC	43°01′57.4″N 75°59′56.2″W
NL F Melick	NLFHM	51°09′24.4″N 6°01′24.6″ E
NL 2 Herten	NLFHM	51°10′37.7″N 5°57′07.1″ E
NL 1 Herten	NLFHM	51°11′00.7″N 5°57′29.1″ E
NL 3 Maarn	NLFHM	52°03′39.6″N 5°22′27.8″ E

After the plants had grown, young fresh leaf tissue was frozen and crushed with liquid nitrogen. The leaf powder was combined with TNE and SDS (5%) and centrifuged. Thereafter isopropanol and ethanol (95%) were added to the supernatant and centrifuged again. DNA samples were analyzed by BGI (Japan) using the RAD‐seq method. RAD‐seq generates genome‐wide sequence data from thousands of orthologous loci with fewer starting materials (Andrews et al. 2016; Shafer et al. 2017). Compared to other methods, RAD‐seq costs relatively less, can provide a large number of reliable molecular markers when no reference genome is available (Lipka et al. 2015), and is often used for demographic analyses (Shafer et al. 2015).

*Stacks* (version 1.27) was used to analyze the SNP calling (Catchen et al. 2013). The program *process_radtags* was used for the demultiplexing and cleaning of the reads. The total number of 75‐bp reads obtained was 282,371,059. The reference genome data for *C. hirsuta* inhabiting Oxford, UK, was downloaded from the website (http://chi.mpipz.mpg.de/index.html) (Gan et al. 2016). Here, we used *BWA MEM* (version 0.7.12) to align the SNP data to the reference genome data. Quality checking was performed using the *Samtools* (version 1.9) *flagstat* program to measure how many raw data reads successfully aligned to the reference genome (Li et al. 2009). After alignment, a total of 278,701,736 reads were mapped to the reference genome, indicating that 98.70% of reads were successfully mapped to the reference genome. Subsequently, *pstacks* and *cstacks* were used to build the loci and create a catalog of the loci and the SNPs identified in all individuals (Catchen et al. 2013). *Sstacks* was used to determine the SNP genotype of each individual after referencing the catalog. The program *populations* was used to compute the genetic statistics and export the output formats for subsequent analysis.

### 2.3 Genetic Diversity and Structure

*ADMIXTURE* software (version 1.3.0) was used to infer the genetic structure among individuals using multilocus genotype data. *ADMIXTURE* uses a maximum‐likelihood approach to jointly estimate allele frequencies in a specified number (*K*) of ancestral populations and the genome‐wide ancestry proportions of each individual (Alexander et al. 2009). Here, we refer to the *K* ancestry components inferred by *ADMIXTURE* as genetic clusters. We used *vcftools* (version 0.1.16) to convert all chromosome names into integers, and subsequently, *plink* software (version 1.9) was used to filter and format the data. Variants with a missing genotype data > 0.1 and the minor allele frequency threshold (MAF) < 0.05 were excluded. Linkage disequilibrium (LD) pruning was performed using *Plink* (version 1.9) to remove highly correlated SNPs prior to genetic structure analyses. To determine the primary number of clusters (*K*), *K* values from 1 to 10 were evaluated using the five‐fold cross‐validation procedure implemented in *ADMIXTURE*. The output provided individual ancestry proportions, with one individual per row (Liu et al. 2020). To assess the model performance and compare clustering solutions across *K* values, cross‐validation (CV) error was calculated (Wang and Zheng 2013). A higher *K* number may lead to over‐fitting (Liu et al. 2020); therefore, we extracted all cross‐validation error values and selected the lowest one as the primary clustering resolution for subsequent analyses. However, results from multiple *K* values (e.g., *K* = 3–6) were also examined to assess the robustness of the inferred genetic structure. Individuals with an ancestry proportion (*Q*) ≥ 0.8 were considered predominantly assigned to a single genetic cluster, whereas individuals with lower values were treated as admixed. Following previous studies (Vähä and Primmer 2006; Steyer et al. 2016; Li, Meegahakumbura, et al. 2024), *Q* ≥ 0.8 was used as a relatively stringent operational criterion for predominant ancestry assignment rather than as a biologically fixed boundary. To evaluate whether individual classification was sensitive to this criterion, we additionally examined assignments using *Q* ≥ 0.7 and *Q* ≥ 0.9, representing less stringent and more stringent thresholds, respectively. The cluster assignments from the selected clustering solution were used for subsequent analyses of geographic distribution and genetic diversity.

Principal component analysis (PCA) was also conducted to assess genetic structure (Patterson et al. 2006). The same filtered SNP dataset used for *ADMIXTURE* was also used for PCA. PCA was performed using *Plink* (version 1.9) and the result was visualized using *R* (version 4.2.3).

Genetic diversity within clusters and genetic differentiation among clusters were calculated using the program in *Stacks* (version 2.68). The following four parameters were calculated: (i) the nucleotide diversity among the loci (*P*_i_); (ii) the expected heterozygosity (*H*_e_); (iii) the observed heterozygosity (*H*_o_); (iv) and the genetic differentiation (*F*_ST_) (Catchen et al. 2013). Standard errors (SE) for *P*_i_, *H*_e_, and *H*_o_ were obtained from the summary statistics generated by *Stacks*. In pairwise *F*_ST_ estimates, 95% confidence intervals were obtained from 10,000 bootstrap replicates by resampling SNPs with replacement. The 2.5th and 97.5th percentiles of the bootstrap distributions were used as the confidence limits.

### 2.4 Species Distribution Model Analysis

To investigate the climate factors associated with the distribution of each genetic cluster in Japan, we used the MaxEnt model combined with ArcGIS (version 10.8), which utilizes presence‐only data and environmental variables to forecast or infer species occurrence (Marcer et al. 2016; Waheed et al. 2023; Putra et al. 2024). The MaxEnt model calculates the maximum entropy distribution likelihood and predicts the possible distribution of the target species under different circumstances based on the maximum entropy of the target species (Muñoz‐Mas et al. 2018; Hallgren et al. 2019). Geographic coordinates (longitude and latitude) were converted into decimal format, and the ArcGIS and SDM tools box (version 2.9) were used to read the climate data and implement operations, such as raster strip and layer stack.

Climate data were obtained from WorldClim (https://www.worldclim.org/data/worldclim21.html), which contained the average monthly climate data for the minimum, mean, and maximum temperatures, precipitation, solar radiation, wind speed, and water vapor pressure for the period 1970 to 2000 within a spatial resolution of 2.5 min (~21 km^2^) (Fick and Hijmans 2017). As *C. hirsuta* germinates in September and dies in June, the climate data for June, July, and August were excluded. The latitude and longitude information from sample sites were loaded to project the coordinates onto the vector map of Japan. The vector map of global climate data was also cropped along the Japan border to obtain a raster map that specifically represents climate data in Japan. Occurrence records for each cluster‐specific model were based on sampling localities at which individuals assigned to the corresponding genetic cluster were detected. The MaxEnt modeling process was repeated 10 times and utilized the cross‐validate method as the replicated running type. Approximately 10,000 background points were randomly sampled by MaxEnt from the whole‐Japan environmental raster domain and were used for all cluster‐specific models. The Jackknife method was used to measure the variable importance. The results obtained included the contribution rate of each environmental factor to the distribution. To evaluate the model's performance, the area under the receiver operating characteristic curve (AUC) was calculated (Ampountolas et al. 2021). These analyses helped characterize climatic conditions that are associated with distribution patterns and provided insights into the potential distribution of these genetic clusters.

Based on the different climate factors that affect the distribution of each genetic cluster, the relationship between the most influential climate factors and their occurrence probability was analyzed. A scatter plot was generated (based on the MAT and occurrence probability in each site) and fitted with a nonlinear regression curve using the log‐normal distribution principle and logistic regression principle to describe general patterns of variation in occurrence probability across temperature gradients.

## 3 Results

### 3.1 Genetic Structure

A total of 11,744 variants were detected in the complete dataset of *C. hirsuta* collected across Japan, the USA, and the NL. After filtering, 251 SNPs were retained for the genetic structure analyses. The overall genotype call rate across all individuals and SNPs in the final filtered dataset was 0.948, and no individuals were excluded due to low genotype call rates. The lowest cross‐validation error was observed at *K* = 4 (0.40228), compared with 0.41114 at *K* = 3, 0.41878 at *K* = 5, and 0.41841 at *K* = 6. Therefore, *K* = 4 was selected as the primary clustering solution for subsequent analyses (Figure 2). At *K* = 4, four ancestry components were inferred by *ADMIXTURE* and were designated Clusters 1–4 (Figure 3A). Sensitivity analysis using *Q* thresholds of 0.7 and 0.9 showed broadly similar patterns of predominant assignment to the four genetic clusters, although individual classifications varied with threshold stringency (Supporting Information Table S1). See Supporting Information S2 and S3 and Figure 3B for genetic structure when *K* is 3, 5, and 6, respectively.

**FIGURE 2:**
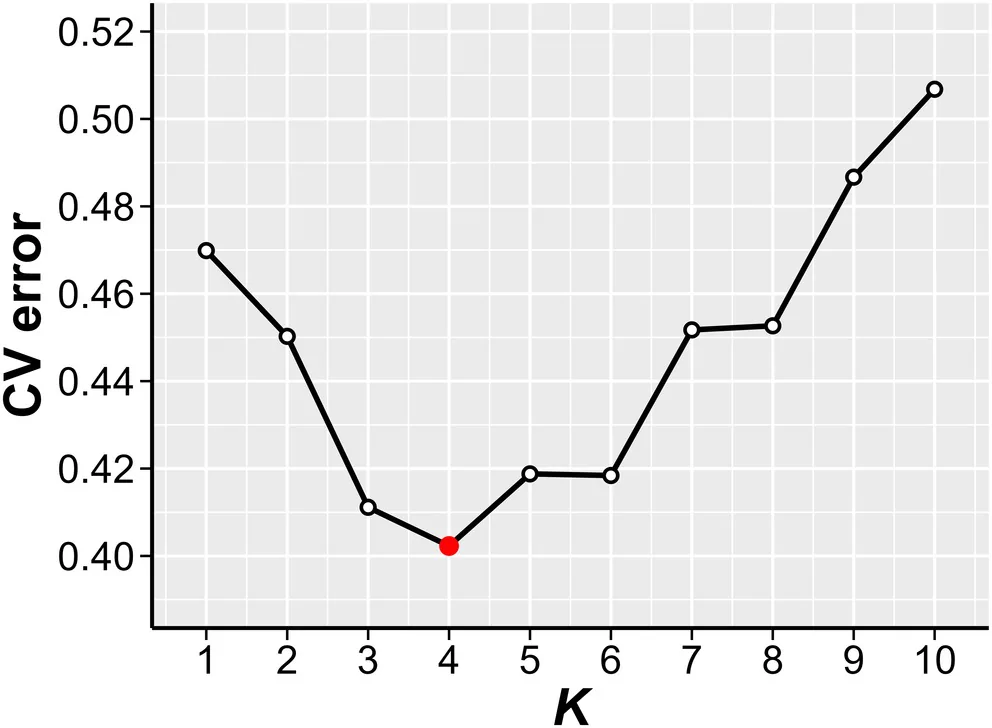
Curve of cross‐validation (CV) error. Plot of *ADMIXTURE* CV error from *K* = 1 to *K* = 10. The solid red point represents the lowest CV error. Based on the minimum CV error, *K* = 4 was chosen for subsequent analyses.

**FIGURE 3:**
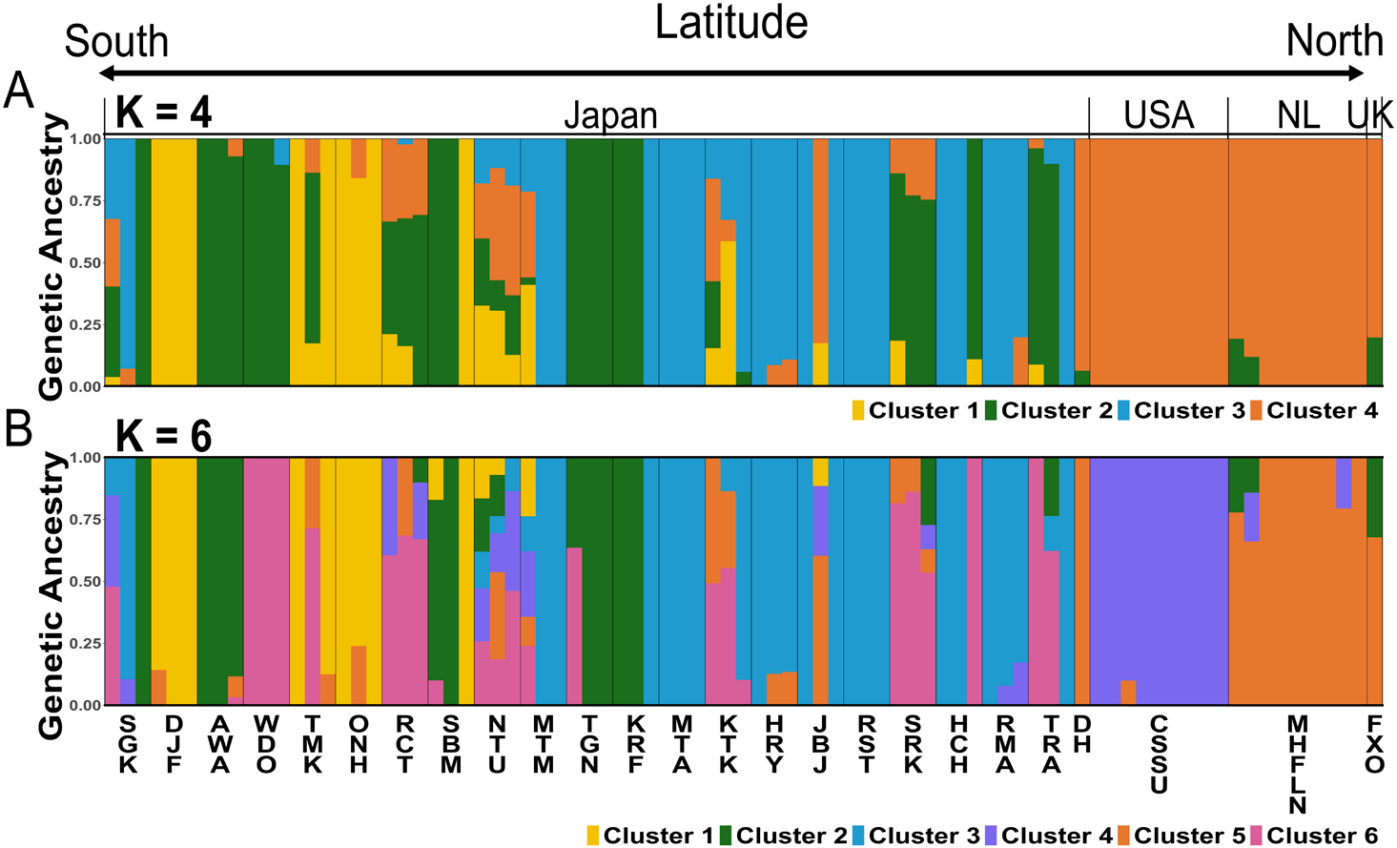
Genetic structure of the *Cardamine hirsuta* individuals inferred by *ADMIXTURE* at *K* = 4 (A) and *K* = 6 (B). Individuals are grouped by sampling location and arranged geographically from south to north. Sampling location abbreviations are listed in Table 1.

Some individuals showed admixed ancestry, indicating contributions from multiple clusters. Based on the cluster assignment criterion described above, 9, 17, 22, and 20 individuals were assigned to Cluster 1, 2, 3, and 4, respectively, while 14 individuals were classified as admixed. Cluster 1, 2, and 3 included the samples from Japan. Cluster 1 was mainly distributed in the southern region of eastern Japan (Figure 4), while Cluster 3 was found in the northern region. Cluster 2 showed a broader distribution across eastern Japan. Cluster 4 included samples from the USA and the NL, as well as two individuals from Japan (Hakodate and Joboji). The reference sample from Oxford, UK, was also assigned to Cluster 4.

**FIGURE 4:**
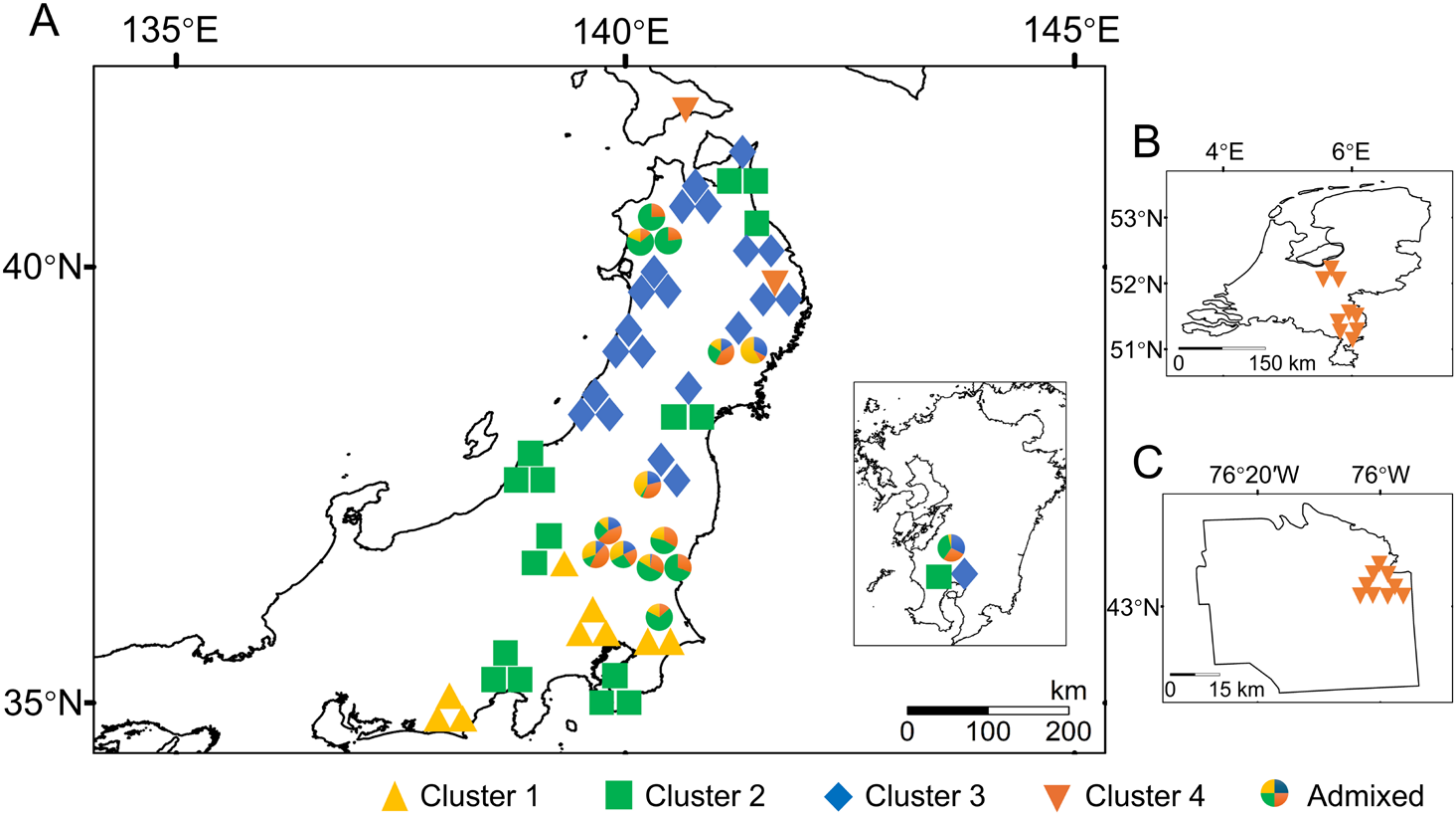
Geographic distribution of genetic clusters based on the *K* = 4 *ADMIXTURE* results (Figure 3). (A) Distribution of genetic clusters in eastern Japan and Kagoshima. (B) Distribution of genetic clusters in the Netherlands. (C) Distribution of genetic clusters in the USA. Symbols represent individuals assigned to each genetic cluster, whereas pie charts indicate admixed individuals.

The first, second, and third axes of the PCA explained 20.20%, 18.58%, and 13.05% of the total genetic variation, respectively. Cluster 4 was separated from others along PC1, while Cluster 3 was distinguishable from others along PC2. Cluster 1 was distinguishable from Cluster 2 along PC3. Admixed individuals were distributed in intermediate positions among clusters, reflecting heterogeneous genetic composition. The PCA results showed partial separation among individuals consistent with the *ADMIXTURE* results, although overlap among clusters was observed (Figure 5).

**FIGURE 5:**
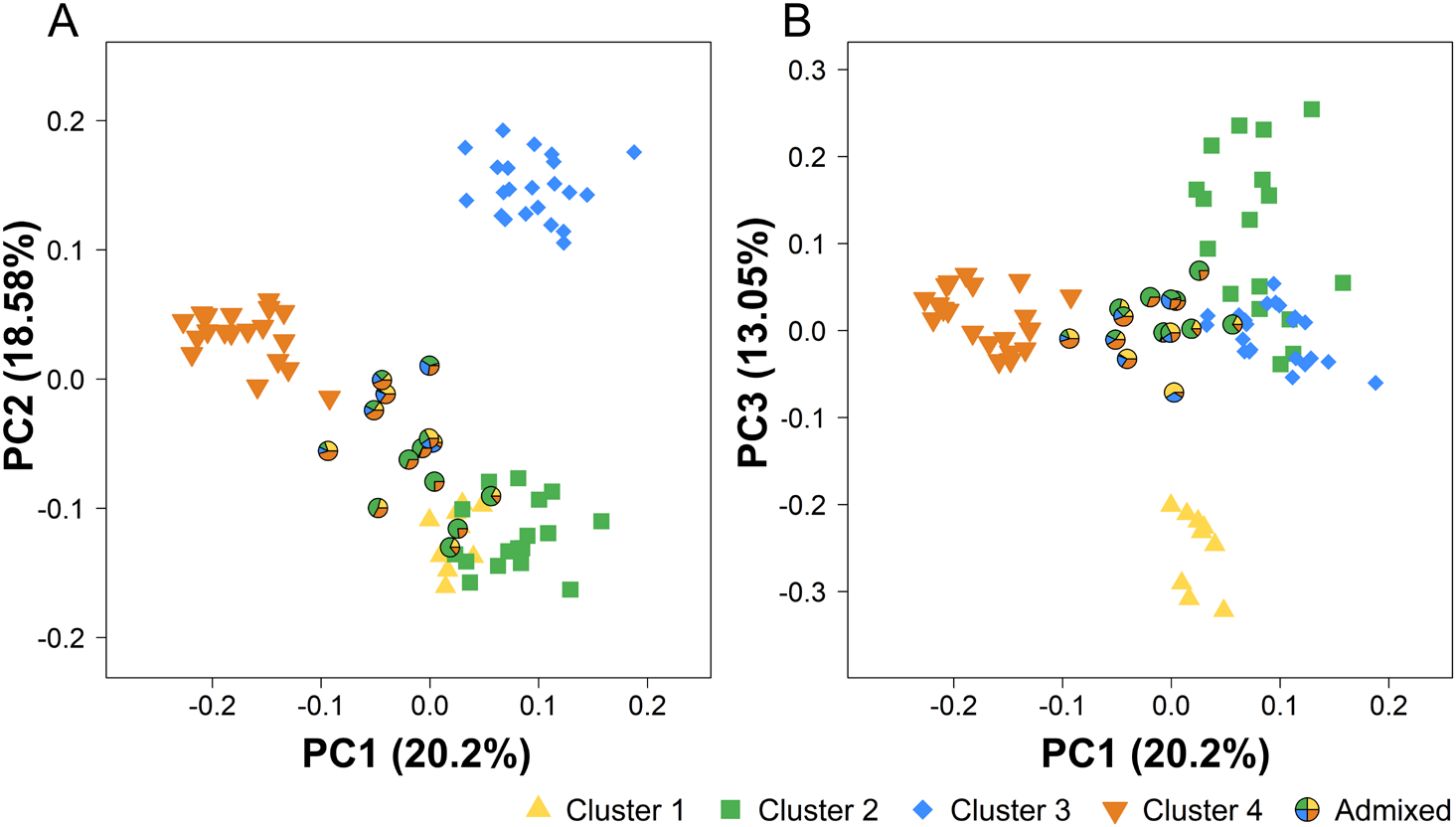
Results of the principal component analysis (PCA) of the *Cardamine hirsuta* individuals. Each symbol represents an individual based on the *K* = 4 *ADMIXTURE* solution. (A) PC1 (20.2%) vs. PC2 (18.58%). (B) PC1 (20.2%) vs. PC3 (13.05%). Four clusters are broadly distinguished, although Cluster 1 shows partial overlap with other groups in PC1–PC2 space.

### 3.2 Genetic Diversity

The nucleotide diversity among the loci (*P*_i_), expected heterozygosity (*H*_e_), observed heterozygosity (*H*_o_), and genetic differentiation (*F*_ST_) were used to evaluate the diversity among the clusters and were estimated using *Stacks* software. As a result, *P*_i_ among the loci ranged from 0.1665 to 0.24266, with the highest observed estimate in Cluster 2 and the lowest in Cluster 1. Similarly, *H*_e_ was the highest in Cluster 2 (0.23302) and the lowest in Cluster 1 (0.15506), whereas Cluster 4 showed the highest *H*_o_ (0.21798) (Table 2). The *F*_ST_ value between Cluster 2 and Cluster 3 was the lowest observed estimate (0.072285, 95% CI = 0.066–0.079), indicating that they had the lowest genetic differentiation, whereas Cluster 1 and Cluster 4 had the highest value (0.151414, 95% CI = 0.137–0.166) (Table 3). Overall, Cluster 2 showed the highest genetic diversity based on *P*_i_ and *H*_e_, whereas Cluster 1 showed the lowest across multiple indices.

**TABLE 2:** Observed heterozygosity (*H*_o_), expected heterozygosity (*H*_e_), and nucleotide diversity among the loci (*P*_i_) of the *Cardamine hirsuta* genetic clusters.

ID	*n*	*H*_o_ (SE)	*H*_e_ (SE)	*P*_i_ (SE)
Cluster 1	9	0.14394 (0.00605)	0.15506 (0.00563)	0.16650 (0.00605)
Cluster 2	17	0.14171 (0.00498)	0.23302 (0.00517)	0.24266 (0.00539)
Cluster 3	22	0.15372 (0.00538)	0.21105 (0.00503)	0.21755 (0.00518)
Cluster 4	20	0.21798 (0.00857)	0.19965 (0.00547)	0.20559 (0.00564)

*Note:* Values in parentheses represent standard errors.

Abbreviations: *H*_e_, expected heterozygosity; *H*_o_, observed heterozygosity; *n*, number of individuals assigned to each genetic cluster; *P*_i_, nucleotide diversity among the loci; SE, standard error.

**TABLE 3:** Genetic differentiation *F*_ST_ among the genetic clusters.

	Cluster1 (*n* = 9)	Cluster 2 (*n* = 17)	Cluster 3 (*n* = 22)	Cluster 4 (*n* = 20)
Cluster 1	—	0.138058 (0.125–0.151)	0.144211 (0.130–0.159)	0.151414 (0.137–0.166)
Cluster 2			0.072285 (0.066–0.079)	0.106110 (0.098–0.114)
Cluster 3				0.106863 (0.097–0.116)
Cluster 4				—

*Note:* Values in parentheses represent 95% confidence intervals based on 10,000 bootstrap replicates.

Abbreviation: *n*, number of individuals assigned to each genetic cluster.

### 3.3 Species Distribution Model Analysis

Results of the MaxEnt analysis indicated differences in climatic associations among the Japanese clusters. Cluster 4 was excluded from the MaxEnt analysis because it was mainly composed of overseas samples and was represented by too few individuals in Japan to allow robust modeling. The distribution of Cluster 1 was suggested to be mainly associated with two climate factors, solar radiation and temperature (Table 4). The potential distribution was present mainly in the central region and coastal areas along the south‐central part of Japan (Supporting Information S4). The distribution of Cluster 3 was suggested to be associated with two climate factors, precipitation and temperature (Table 4). The primary potential distribution region was in the northeastern region of Japan, with possible occurrences in the central region, southern Japan, and Hokkaido (Supporting Information S6). The distribution of Cluster 2 was related to four climate factors: temperature, precipitation, solar radiation, and wind speed (Table 4). The potential distribution region was more frequent in the central region, northeastern region, and southern regions, which suggests a relatively high probability of a nationwide presence (Supporting Information S5).

**TABLE 4:** Climate factor percentage contribution among the genetic clusters in Japan.

Cluster	Climate factor	Percentage contribution (%)
Cluster 1	Solar radiation in January	37.7
	Maximum temperature in April	12.2
	Solar radiation in September	9.6
	Solar radiation in October	8.7
Cluster 2	Maximum temperature in September	54.0
	Precipitation in September	15.7
	Solar radiation in September	7.0
	Wind speed in May	7.0
Cluster 3	Precipitation in September	51.7
	Minimum temperature in February	35.1

Because temperature‐related variables contributed to all genetic clusters, we analyzed the relationship between the occurrence probability and MAT for every grid in Japan from 20°25′ N to 45°31′ N, and the potential distribution of each cluster was fitted using nonlinear regression curves (Figure 6). The results suggest that Cluster 3 showed higher predicted occurrence probabilities under cooler areas, whereas Cluster 1 showed higher predicted occurrence probabilities under relatively warmer areas. Cluster 2 had a relatively broad range of potential distribution compared to Cluster 1 and Cluster 3. The modeled potential distributions of the three clusters broadly overlapped, and no obvious gap was detected along the temperature gradient in which all three clusters showed low predicted occurrence probabilities. Admixed individuals were found in the temperature region where the potential distribution of the three clusters overlap.

**FIGURE 6:**
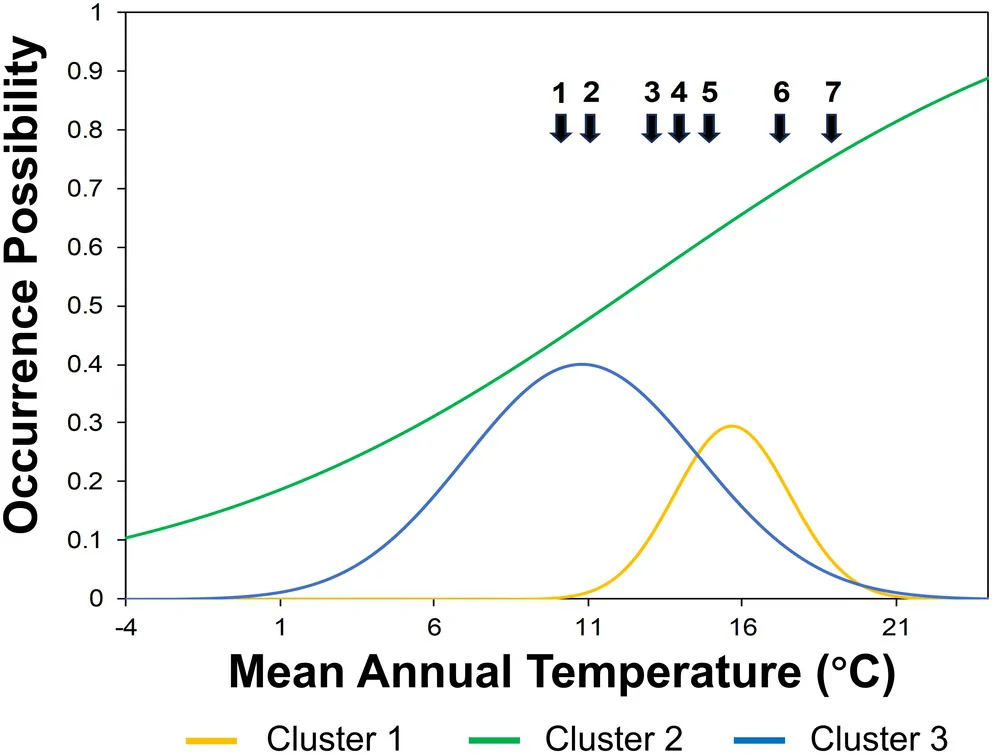
Occurrence probability as a function of mean annual temperature (MAT) across all raster grid cells within the whole‐Japan MaxEnt projection domain (20°25′ N–45°31′ N). This geographic range represents the MaxEnt projection domain area. Numbers 1–7 indicate locations where admixed individuals were detected: 1 Kuroishi, 2 Kitakami, 3 Motomiya, 4 Utsunomiya, 5 Tsuchiura, 6 Kimitsu, and 7 Kagoshima.

## 4 Discussion

In this study, we revealed substantial genetic structure in *C. hirsuta* across eastern Japan based on genome wide SNP data, with genetic clusters showing contrasting geographic and climatic distributions and individuals with mixed ancestry occurring mainly where cluster distributions overlapped. We discuss these patterns in relation to the three hypotheses proposed in the Introduction.

### 4.1 A Genetic Cluster With a Broad Environmental Distribution

Hypothesis 1 proposed that a genetic cluster can occur across a wide range of environmental conditions. Our results were consistent with this hypothesis because Cluster 2 occurred across a broader geographic range and showed a broader modeled temperature range of potential distribution than Cluster 1 and 3. This contrasts with previous studies showing that such broad distributions of a single species are generally associated with genetic and functional differentiation along environmental gradients (e.g., Ishikawa et al. 2007; Machino et al. 2021; Ishizuka et al. 2020; Yoshida et al. 2023). For example, the temperature dependence of the photosynthetic rates and nitrogen allocation between photosynthetic proteins differ between Hokkaido, Sendai, and Shizuoka in the native species *Plantago asiatica* (Ishikawa et al. 2007). However, our results suggest that one genetic group may occur across a wide range of environmental variations.

One might consider that Cluster 2 contains finer‐scale genetic substructure associated with different temperature conditions. Because the cross‐validation errors for neighboring values of *K* were relatively similar, we also examined whether the broad distribution of Cluster 2 depended on the clustering resolution. Although *K* = 4 had the lowest cross‐validation error (0.40228), the cross‐validation error at *K* = 6 was only slightly higher (0.41841). In fact, when we assumed *K* = 6, Cluster 2 in *K* = 4 was subdivided into two ancestry components (Cluster 2 and Cluster 6 in *K* = 6; Figure 3B). Such subdivision at a higher value of *K* may reflect finer‐scale or hierarchical genetic structure rather than indicating a uniquely “true” number of genetic clusters (Verity and Nichols 2016; Lawson et al. 2018). However, Cluster 6 in *K* = 6 was also distributed in a wide range of latitude, suggesting that even the sub‐cluster was distributed across a wide range of temperature conditions. Nevertheless, the present distributional and MaxEnt analyses do not directly demonstrate physiological tolerance or adaptation to a broad temperature regime. Whether the broad climatic distribution of this genetic group reflects physiological traits, phenotypic plasticity, or other ecological mechanisms requires direct experimental evaluation.

### 4.2 Climatic Differentiation Among Genetic Clusters

The distributions of Cluster 1 and Cluster 3 were consistent with Hypothesis 2, which proposed that different genetic clusters are distributed across distinct environmental conditions. Cluster 1 predominantly occurred in southern regions of eastern Japan, while Cluster 3 was mainly distributed in northern regions of eastern Japan. The cluster‐specific distribution models were also consistent with geographic differentiation, with Cluster 1 showing stronger associations with relatively warmer conditions and Cluster 3 with cooler conditions. These contrasting distribution patterns suggest that different genetic clusters occupy different portions of the environmental gradient, which may partly contribute to the overall distribution of *C. hirsuta* across a wide range of conditions.

### 4.3 Admixture and Overlap Among Genetic Clusters

In the third hypothesis, we considered that individuals with mixed ancestry may fill distributional gaps between different original genetic clusters. Although individuals with mixed ancestry were detected across our study sites, the modeled potential distributions of the genetic clusters broadly overlapped along the temperature gradient and did not reveal an obvious gap. Thus, the specific gap‐filling pattern predicted by Hypothesis 3 was not supported by our results. Instead, individuals with mixed ancestry often appear in regions where the geographical distributions of genetic clusters overlap. The MaxEnt results also indicated that admixed individuals were more likely to appear under conditions where multiple genetic clusters showed high occurrence probability simultaneously. These results suggest a close spatial association between mixed ancestry and regions of cluster overlap. However, this pattern does not exclude the possibility that admixture may have ecological or adaptive consequences. Whether individuals with mixed ancestry differ in fitness or environmental responses from predominantly assigned individuals requires direct experimental evaluation. In future, admixed individuals coexist with individuals from original clusters if they have similar fitness to original individuals in the area, while admixed individuals may exclude original individuals if they have higher fitness.

### 4.4 Genetic Structure and Diversity

*C. hirsuta* is native to Europe (Yatsu et al. 2003), and all individuals sampled from the NL and the USA in this study were predominantly assigned to Cluster 4. In contrast, Cluster 4 was rare among our samples from eastern Japan, with only two individuals, from Joboji and Hakodate, predominantly assigned to this cluster. Cluster 4 ancestry proportion was also detected in many admixed individuals. There are a few possibilities to explain this pattern: (a) according to the study of Matsuhashi et al. (2016), there is another large population in western Japan (west group), which was not identified in our study. If the west group is genetically related to Cluster 4, one possibility is that some of its individuals migrated from western to eastern Japan. (b) Previously, Cluster 4 ancestry may have inhabited eastern Japan but was subsequently declined by other genetic clusters. (c) Cluster 4 may have been introduced into eastern Japan relatively recently, followed by genetic mixing with other genetic clusters already present in the region. Because *C. hirsuta* is a winter annual plant, genetic mixing could potentially occur over a relatively short time period following contact among genetic clusters. Distinguishing among these scenarios will require broader genomic sampling from western Japan and the European native range, as well as additional sampling from introduced populations outside Japan.

Genetic diversity estimates also differed numerically among genetic clusters. Cluster 2, which showed the broadest geographic distribution among the major Japanese clusters, had the highest observed nucleotide diversity and expected heterozygosity, whereas Cluster 1 showed lower values. Genetic diversity can provide variation on which selection acts and may therefore contribute to the evolutionary potential of invasive populations. Thus, the relatively high diversity of Cluster 2 and lower diversity of Cluster 1 are consistent with their broader and more restricted distributions, respectively, although the present data do not establish that differences in genetic diversity caused these contrasting distributional patterns. Relatively low genetic differentiation among clusters (especially between Cluster 2 and Cluster 3), together with the occurrence of individuals with mixed ancestry, further suggests that the clusters are not completely genetically isolated.

Previously, the genetic structure of *C. hirsuta* in Japan was studied by Matsuhashi et al. (2016) using microsatellite markers. In their results, most *C. hirsuta* individuals were categorized into three groups, including north, east, and west groups. Our study sites overlapped with the distribution of the north and east groups, and the distribution of the east group appears similar to that of Cluster 1. On the other hand, the geographic distribution of the north group reported by Matsuhashi et al. (2016) broadly overlaps with the combined distributions of Clusters 2 and 3 in our study, suggesting that the region corresponding to the north group reported by Matsuhashi et al. (2016) consists of two genetic clusters. This discrepancy may be explained by the higher genetic resolution of our method (RAD‐seq) compared with the microsatellite markers used by Matsuhashi et al. (2016). The relationship between the west group and the genetic clusters identified in our study could not be evaluated because we lacked samples from western and southwestern Japan. In our study, admixed individuals were primarily detected in the central to northern parts of eastern Japan. This pattern is partially consistent with the findings of Matsuhashi et al. (2016), who reported admixture in both the Kansai region and the central to northern parts of eastern Japan.

### 4.5 Limitations and Future Perspectives

Since *C. hirsuta* was first recorded in Japan in the 1970s, its current distribution likely represents a non‐equilibrium phase of invasion. The coexistence of multiple clusters and the presence of mixed ancestry indicate ongoing or recent genetic mixing among clusters, while their geographic distributions may continue to change as the invasion proceeds. Also, sampling from the native range was limited, which constrains inference of introduction sources and the invasion history of the genetic clusters, particularly Cluster 4. In addition, the relatively small and uneven sample sizes among genetic clusters, particularly for Cluster 1, should be considered when interpreting the cluster‐specific MaxEnt models. These models describe potential distributions and climatic associations of the sampled genetic clusters, but do not directly demonstrate physiological tolerance or adaptation to particular environmental conditions (Hernandez et al. 2006; Pearson et al. 2007). Therefore, we need to carefully interpret the environmental niche predicted by the MaxEnt model. Besides, one possibility is that some individuals of a genetic cluster may inhabit an area where their fitness is not necessarily very high. Such individuals may be replaced by better adapted individuals or other species in the future. Temporal changes in the genetic structure of invasive species may deepen our understanding of long‐term responses of plants to environmental changes.

## 5 Conclusion

In this study, we found that *C. hirsuta* in eastern Japan is composed of multiple genetic clusters with different distributions and climatic associations. Two clusters were distributed in narrow environmental conditions, where one inhabits cooler regions, while the other is distributed in warmer regions. Another cluster had a relatively broad geographic and climatic distribution. In addition, admixture among clusters was observed, particularly in regions where the distributions of different genetic clusters overlapped. Our results demonstrate that the broad distribution of *C. hirsuta* in eastern Japan is associated with both the coexistence of genetically differentiated clusters showing contrasting climatic associations and the occurrence of a genetic cluster across a comparatively broad climatic range. The specific gap‐filling pattern predicted for individuals with mixed ancestry was not supported; instead, these individuals occurred mainly in regions where the distributions of genetic clusters overlapped. Our findings highlight that the coexistence of clusters occupying narrow and wide environmental ranges may contribute to broad species distribution. From an ecological and practical perspective, considering such intraspecific genetic structure may also be important when assessing the spread of invasive species. This is because genetic clusters with contrasting climatic associations and distributional breadth may differ in their potential for further spread. Incorporating such genetic information into invasion assessments may therefore improve the identification of areas with potential for range expansion and support more effective monitoring of ongoing invasions.

## Author Contributions

**Bo O. Zhang:** conceptualization (equal), data curation (equal), formal analysis (lead), methodology (equal), validation (equal), visualization (lead), writing – original draft (lead), writing – review and editing (equal). **Yiheng Shen:** data curation (equal), formal analysis (equal), investigation (equal), methodology (equal), writing – original draft (equal), writing – review and editing (equal). **Hiroshi Ozaki:** data curation (equal), formal analysis (equal), investigation (lead), methodology (lead), writing – review and editing (equal). **Naofumi Yoshida:** data curation (supporting), formal analysis (supporting), methodology (supporting), writing – review and editing (equal). **Shin‐Ichi Morinaga:** conceptualization (equal), data curation (supporting), formal analysis (supporting), writing – review and editing (equal). **Onno Muller:** resources (equal), writing – review and editing (equal). **Kouki Hikosaka:** conceptualization (lead), funding acquisition (lead), investigation (equal), project administration (lead), resources (lead), supervision (lead), writing – original draft (equal), writing – review and editing (lead).

## Funding

This study was supported by JST SPRING Grant Number JPMJSP2114 and was partly supported by JST CREST Grant Number JPMJCR11B3 as well as KAKENHI (No. 25291095, 17H03727, 20H03317, and 23H02549) from MEXT, Japan.

## Conflicts of Interest

The authors declare no conflicts of interest.

## Supporting information


**Figure S1:** ece374480‐sup‐0001‐Supinfo.docx. *Cardamine hirsuta* observed in the field in Japan.
**Figure S2:** Genetic structure of the *Cardamine hirsuta* individuals inferred by *ADMIXTURE* at *K* = 3.
**Figure S3:** Genetic structure of the *Cardamine hirsuta* individuals inferred by *ADMIXTURE* at *K* = 5.
**Table S1:** Sensitivity of individual genetic‐cluster classification to alternative *ADMIXTURE* thresholds.
**Figure S4:** Potential distribution and climate factor limitations for the Cluster 1 (*K* = 4).
**Figure S5:** Potential distribution and climate factor limitations for the Cluster 2 (*K* = 4).
**Figure S6:** Potential distribution and climate factor limitations for the Cluster 3 (*K* = 4).

## Data Availability

The raw sequencing data generated in this study have been deposited in the DDBJ Sequence Read Archive (DRA) under BioProject accession PRJDB42445 and run accessions DRR1058032–DRR1058113 (https://ddbj.nig.ac.jp/search/entry/bioproject/PRJDB42445/).
