# Supplementary material for: Genetic Structure Along Temperature Gradient in a Recent Invader Cardamine hirsuta in Eastern Japan
Source: Ecol Evol. 2026 Oct 9;16(10):e74480. doi: 10.1002/ece3.74480 (PMC13652653; doi:10.1002/ece3.74480)
Supplement: Supplementary file 1 [file ECE3-16-e74480-s001.docx]

**Supplementary Information**

Genetic structure along temperature gradient in a recent invader *Cardamine hirsuta* in eastern Japan

Bo O. Zhang^1,*^, Yiheng Shen^1^, Hiroshi Ozaki^2^, Naofumi Yoshida^1^, Shin-Ichi Morinaga^3^, Onno Muller^4^, Kouki Hikosaka^1,*^

^1^ Graduate School of Life Sciences, Tohoku University, Aoba, Sendai 908-8578, Japan

^2^ Translational Research Support Office, National Cancer Center Hospital East, Kashiwa 277-8577, Japan

^3^ Department of Natural & Environmental Science, Teikyo University of Science, Adachi, Tokyo 120-0045, Japan

^4^ Institute for Bio-and Geosciences, IBG-2: Plant Sciences, Research Center Jülich GmbH, Wilhelm-Johnen-Strasse, Jülich 52428, Germany

* Corresponding authors:

Bo O. Zhang: zhang.bo.p3@dc.tohoku.ac.jp

Kouki Hikosaka: hikosaka@tohoku.ac.jp

**Fig. S1**

**Fig. S1** Cardamine hirsuta observed in the field in Japan

**Fig. S2**

**Fig. S2** Genetic structure of the *Cardamine hirsuta* individuals inferred by *ADMIXTURE* at *K* = 3. Individuals are grouped by sampling location and arranged geographically from south to north. Sampling location abbreviations are listed in Table 1.

**Fig. S3**

**Fig. S3** Genetic structure of the *Cardamine hirsuta* individuals inferred by *ADMIXTURE* at *K* = 5. Individuals are grouped by sampling location and arranged geographically from south to north. Sampling location abbreviations are listed in Table 1.

**Table S1 Sensitivity of individual genetic-cluster classification to alternative *ADMIXTURE* thresholds.**

| **Threshold** | **Cluster 1** | **Cluster 2** | **Cluster 3** | **Cluster 4** | **Admixed** | **Total** |
| --- | --- | --- | --- | --- | --- | --- |
| **≥ 0.7** | **9** | **19** | **22** | **20** | **12** | **82** |
| **≥ 0.8** | **9** | **17** | **22** | **20** | **14** | **82** |
| **≥ 0.9** | **8** | **13** | **20** | **17** | **24** | **82** |

**Table S1** Individuals were considered predominantly assigned to a genetic cluster when their ancestry proportion (*Q*) for that cluster was equal to or greater than the specified threshold; otherwise, they were classified as admixed. *Q* ≥ 0.8 was used as the primary criterion, whereas *Q* ≥ 0.7 and *Q* ≥ 0.9 were examined as less stringent and more stringent sensitivity thresholds, respectively.

**Fig. S4**

**Fig. S4** Potential distribution and climate factor limitations for the Cluster 1 (*K*=4). Figure a represents the AUC (the Area Under the Receiver operating characteristic Curve) of Cluster 1. Figure b represents the possibility of the presence of Cluster 1 plants as a function of climate environmental factors. The figure c represents Cluster 1 potential distribution and the possible occurrence probabilities in various places are represented by different colors.

**Fig. S5**

**Fig. S5** Potential distribution and climate factor limitations for the Cluster 2 (*K*=4). Figure a represents the AUC (the Area Under the Receiver operating characteristic Curve) of Cluster 2. Figure b represents the possibility of the presence of Cluster 2 plants as a function of climate environmental factors. The figure c represents Cluster 2 potential distribution and the possible occurrence probabilities in various places are represented by different colors.

**Fig. S6**

**Fig. S6** Potential distribution and climate factor limitations for the Cluster 3 (*K*=4). Figure a represents the AUC (the Area Under the Receiver operating characteristic Curve) of Cluster 3. Figure b represents the possibility of the presence of Cluster 3 plants as a function of climate environmental factors. Figure c represents Cluster 3 potential distribution and the possible occurrence probabilities in various places are represented by different colors.
